# Characterization of adipose tissue macrophages and adipose-derived stem cells in critical wounds

**DOI:** 10.7717/peerj.2824

**Published:** 2017-01-04

**Authors:** Bong-Sung Kim, Pathricia V. Tilstam, Katrin Springenberg-Jung, Arne Hendrick Boecker, Corinna Schmitz, Daniel Heinrichs, Soo Seok Hwang, Jan Philipp Stromps, Bergita Ganse, Ruedger Kopp, Matthias Knobe, Juergen Bernhagen, Norbert Pallua, Richard Bucala

**Affiliations:** 1 Plastic and Reconstructive Surgery, Hand Surgery—Burn Center, Rheinisch-Westfälische Technische Hochschule Aachen, Aachen, Germany; 2 Department of Medicine, Yale University, New Haven, United States; 3 Institute of Biochemistry and Molecular Cell Biology, Rheinisch-Westfälische Technische Hochschule Aachen, Aachen, Germany; 4 Department of Immunology, Yale University, New Haven, United States; 5 Department of Orthopedic Trauma Surgery, Rheinisch-Westfälische Technische Hochschule Aachen, Aachen, Germany; 6 Department of Intensive Care Medicine, Rheinisch-Westfälische Technische Hochschule Aachen, Aachen, Germany; 7 Department of Vascular Biology, Institute for Stroke and Dementia Research, Ludwig-Maximilians-Universität München (LMU), Munich, Germany; 8 Munich Cluster for Systems Neurology (SyNergy), Ludwig-Maximilians-Universität München (LMU), Munich, Germany

**Keywords:** Wound repair, Adipose tissue, Inflammation, Macrophages, Adipose-derived stem cells, Polarization, M2, M1

## Abstract

**Background:**

Subcutaneous adipose tissue is a rich source of adipose tissue macrophages and adipose-derived stem cells which both play a key role in wound repair. While macrophages can be divided into the classically-activated M1 and the alternatively-activated M2 phenotype, ASCs are characterized by the expression of specific stem cell markers.

**Methods:**

In the present study, we have investigated the expression of common macrophage polarization and stem cell markers in acutely inflamed adipose tissue. Subcutaneous adipose tissue adjacent to acutely inflamed wounds of 20 patients and 20 healthy subjects were harvested and underwent qPCR and flow cytometry analysis.

**Results:**

Expression levels of the M1-specific markers CD80, iNOS, and IL-1b were significantly elevated in inflammatory adipose tissue when compared to healthy adipose tissue, whereas the M2-specific markers CD163 and TGF-*β* were decreased. By flow cytometry, a significant shift of adipose tissue macrophage populations towards the M1 phenotype was confirmed. Furthermore, a decrease in the mesenchymal stem cell markers CD29, CD34, and CD105 was observed whereas CD73 and CD90 remained unchanged.

**Discussion:**

This is the first report describing the predominance of M1 adipose tissue macrophages and the reduction of stem cell marker expression in acutely inflamed, non-healing wounds.

## Introduction

Wound healing disorders remain a challenge for specialists and the burden to global healthcare systems is substantial (Mudge, 2015). In the multifactorial process of wound healing, adipose tissue located in the subcutaneous tissue layers adjacent to the wounds plays a critical role as a dynamic organ secreting soluble factors and as a reservoir for regenerative cells (Kim et al., 2015a).

Although mature adipocytes account for the majority of adipose tissue mass, two main regulators of body homeostasis and also wound repair are resident macrophages and stem cells (Hassan, Greiser & Wang, 2014; Koh & DiPietro, 2011) Adipose tissue macrophages (ATM) are the largest leukocyte fraction in adipose tissue, comprising approximately 10% of the total cell number in lean subjects (Kraakman et al., 2014). Adipose tissue is also a rich source of stem cells—the adipose-derived stem cells (ASC) which show considerable plasticity as they are able to undergo multilineage differentiation (Zuk et al., 2001).

The lion’s share of current adipose tissue research is directed at investigation of the chronic adipose tissue inflammation that accompanies obesity and insulin resistance. In 2003, two groups independently reported that there is an accumulation of ATMs in obese rodents and humans and suggested an association between ATMs and insulin resistance (Weisberg et al., 2003; Xu et al., 2003). Dysfunctional ATMs appear to play a key role in the maintenance of chronic adipose tissue inflammation and different classifications were introduced to better characterize macrophage physiology in disease. The “M1/M2-polarization” of macrophages has become a widely accepted nomenclature to categorize macrophages into subpopulations with distinct functions during the inflammatory response. “Classically activated” M1 macrophages are considered the pro-inflammatory subtype whereas “alternatively activated” M2 macrophages are known to possess anti-inflammatory properties (Sica & Mantovani, 2012). It was found that ATMs show altered macrophage subsets in obesity, and ATMs in obese subjects were skewed towards the M1 phenotype (Lumeng, Bodzin & Saltiel, 2007).

ASCs are multipotent mesenchymal stem cells resident in adipose tissue. It was reported that the yield of ASCs is 100–500 times higher when compared bone marrow-derived stem cells (BMSC) (Casteilla & Dani, 2006; D’Andrea et al., 2008). As ASCs can be abundantly harvested by liposuction which is an accepted, safe, and routinely performed procedure, they are considered a ready stem cell source for applications in regenerative medicine and particularly in wound repair (Ugarte et al., 2003).

Studies so far failed to characterize the changes in ATMs and ASCs in subcutaneous adipose tissue which is the tissue layer in immediate proximity to skin and thus plays an important, yet underestimated role during wound repair (Campbell et al., 2010; Sugihara et al., 2001). In the present study, we examined the polarization of ATMs and the expression of common stem cell markers in adipose tissue collected from subcutaneous tissue layers adjacent to acutely inflamed, non-healing wounds. We investigated both cell types as there is a dynamic interplay between ASCs and ATMs which also effects wound repair. ASCs are able to influence the phenotype of ATMs whereas ATMs control differentiation, proliferation, migration of ASCs (Shang et al., 2015; Sorisky, Molgat & Gagnon, 2013). Consequently, we hypothesized that a combination of a pathological dysbalance of ATM subtypes and a phenotypic change of ASCs leads to the progression of in inflamed non-healing wounds.

## Patients and Methods

Inflammatory adipose tissue (IAT) samples were collected from patients with wound healing disorders and healthy patients with normal adipose tissue (HAT) independent of their BMI as reported earlier (Kim et al., 2015b.). The group of wound healing disorders (Table 1) included 20 patients (10 male and 10 female, mean age: 52.75 ± 2.361 year; body mass index (BMI): 28.05 ± 0.96 kg/m^2^). The group of healthy controls (Table 2) was age and BMI-matched (10 male and 10 female, mean age: 55.85 ± 3.8 years; mean BMI: 28.1 ± 1.3 kg/m^2^). Wound healing disorders were defined as wounds caused by external trauma or after surgical intervention that were not resolved within a period of four weeks after trauma (Izadi & Ganchi, 2005; Leaper & Durani, 2008). Furthermore, wounds showed classic signs of local inflammation (tumor, calor, rubor, dolor and functio laesa) and negative bacterial swab samples at the time of tissue harvest. Small blocks of adipose tissue were excised within a radius of 1 cm to the wound. The control group contained healthy patients who underwent elective plastic surgery (e.g., fat reduction surgeries, flap thinning procedures).

**Table 1:** List of inflammatory adipose tissue samples.

Number	Gender	Age (years)	BMI (kg/m^2^)	Specification
1	m	40	32	Postoperative wound healing disorder
2	w	49	25	Postoperative wound healing disorder
3	m	40	32	Postoperative wound healing disorder
4	m	42	20	Wound healing disorder after external trauma
5	w	74	37	Wound healing disorder after external trauma
6	w	39	35	Postoperative wound healing disorder
7	w	38	35	Wound healing disorder after external trauma
8	m	48	21	Postoperative wound healing disorder
9	w	71	19	Wound healing disorder after external trauma
10	w	64	22	Wound healing disorder after external trauma
11	m	62	27	Wound healing disorder after external trauma
12	m	62	34	Wound healing disorder after external trauma
13	w	70	26	Wound healing disorder after external trauma
14	w	52	25	Wound healing disorder after external trauma
15	m	69	28	Postoperative wound healing disorder
16	m	70	34	Postoperative wound healing disorder
17	w	53	25	Wound healing disorder after external trauma
18	m	76	35	Wound healing disorder after external trauma
19	m	83	27	Wound healing disorder after external trauma
20	w	15	23	Postoperative wound healing disorder

**Table 2:** List of healthy adipose tissue samples.

Number	Gender	Age (years)	BMI (kg/m^2^)
1	m	54	26
2	w	62	29
3	w	43	31
4	w	46	24
5	m	65	27
6	w	47	22
7	m	57	29
8	w	44	23
9	m	51	29
10	m	51	29
11	w	61	34
12	m	61	33
13	w	43	37
14	w	63	23
15	m	51	35
16	w	64	29
17	w	56	25
18	m	20	24
19	m	59	27
20	m	57	25

Samples were immediately minced. Blood vessels, connective tissue, and importantly also necrotic tissue were removed and samples were washed with phosphate-buffered saline (PBS). One part of the sample was stored at −80 °C for later quantitative real-time polymerase chain reaction (qRT-PCR) analysis. Another part was immediately used for flow cytometric analysis as described below. All surgeries were performed in the Department of Plastic Surgery, Hand Surgery–Burn Center of the RWTH University Hospital Aachen, Germany and approved by the local ethics committee (Ethikkommission RWTH Aachen, EK 163/07). Patients provided written consent and experiments were performed in compliance with the Declaration of Helsinki Principles.

### Hematoxylin/eosin (HE) staining

Adipose tissue samples were collected and fixed in 4% paraformaldehyde. After dehydration, the tissue was embedded in paraffin. Sections were cut by a microtome and standard HE staining was performed.

### Isolation of the stromal vascular fraction (SVF) and cell counting

Adipose tissue was digested with collagenase solution (1% bovine serum albumin (BSA), 0.2% collagenase (type I; Worthington Biochemical Corp., NJ, USA)) for 45 min at 37 °C under constant shaking. Collagenase digestion leads to the separation of adipocytes and the SVF which represents the non-buoyant cellular fraction that also contains ATMs and ASCs. Digested tissue was filtered through a 250 µm mesh and centrifuged at 300 g for ten minutes. After discarding the supernatant, the SVF-containing pellet was resuspended in erythrocyte lysis buffer for five minutes. The reaction was stopped by adding PBS and the solution was centrifuged again at 300 g for ten minutes. Cells were counted in a standard hemocytometer and dead cells were excluded by trypan blue staining.

### Isolation of messenger RNA (mRNA), reverse transcription and quantitative real-time polymerase chain reaction (qRT-PCR)

Messenger RNA from adipose tissue was isolated by the QIAzol Lysis Reagent (Qiagen NV, Venlo, Netherlands) following the manufacture’s instructions. mRNA was reverse-transcribed into cDNA by the First Strand cDNA Synthesis Kit (Thermo Fisher Scientific Inc, Waltham, MA, USA) following the manufacture’s instructions. qRT-PCR was carried out using the 2× SensiMix SYBR No-ROX Kit (Peqlab Biotechnology, Erlangen, Germany) on a RotorGene 6000 (Qiagen NV, Venlo, Netherlands). Glyceraldehyde 3-phosphate dehydrogenase (GAPDH) was used as the housekeeping gene. Primers used for qRT-PCR are found in Table 3.

### Flow cytometry

SVF cells were permeabilized with ice-cold methanol and surface stained with CD80-PE, and CD163-APC, and intracellularly stained with CD68-eFluor450. All antibodies were purchased from eBioscience (Frankfurt am Main, Germany). Flow cytometry was performed on a FACS Canto-II (BD Bioscience, Heidelberg, Germany) and data was analyzed with FlowJo (version v10.1r1; FlowJo, Ashland, OR, USA).

**Table 3:** List of used qRT-PCR primer.

Gene	Forward (5′–3′)	Reverse (5′–3′)
GAPDH (69)	TGGTATCGTGGAAGGACTCATGAC	ATGCCAGTGAGCTTCCCGTTCAGC
CD80 (70)	CTGCCTGACCTACTGCTTTG	GGCGTACACTTTCCCTTCTC
CD163 (70)	ACATAGATCATGCATCTGTCATTTG	ATTCTCCTTGGAATCTCACTTCTA
IL-1*β* (71)	GCACGATGCACCTGTACGAT	CACCAAGCTTTTTTGCTGTGAGT
IL1-RA (72)	GCGAGAACAGAAAGCAGGAC	CCTTCGTCAGGCATATTGGT
iNOS (73)	ATGCCCGATGGCACCATCAGA	TCTCCAGGCCCATCCTCCTGC
TGF-*β* (74)	CCCAGCATCTGCAAAGCTC	GTCAATGTACAGCTGCCGCA

### Statistical analysis

GraphPad Prism (GraphPad Software, Inc., La Jolla, CA, USA) was used for statistical analysis. All data are expressed as mean ± SEM. As the data was not normally distributed as calculated by the Shapiro–Wilk normality test, we performed a non-parametrical Mann–Whitney test to identify significant differences with a *p* value of <0.05 considered as significant.

## Result

### IAT shows increased infiltration of inflammatory cells

**Figure 1:**
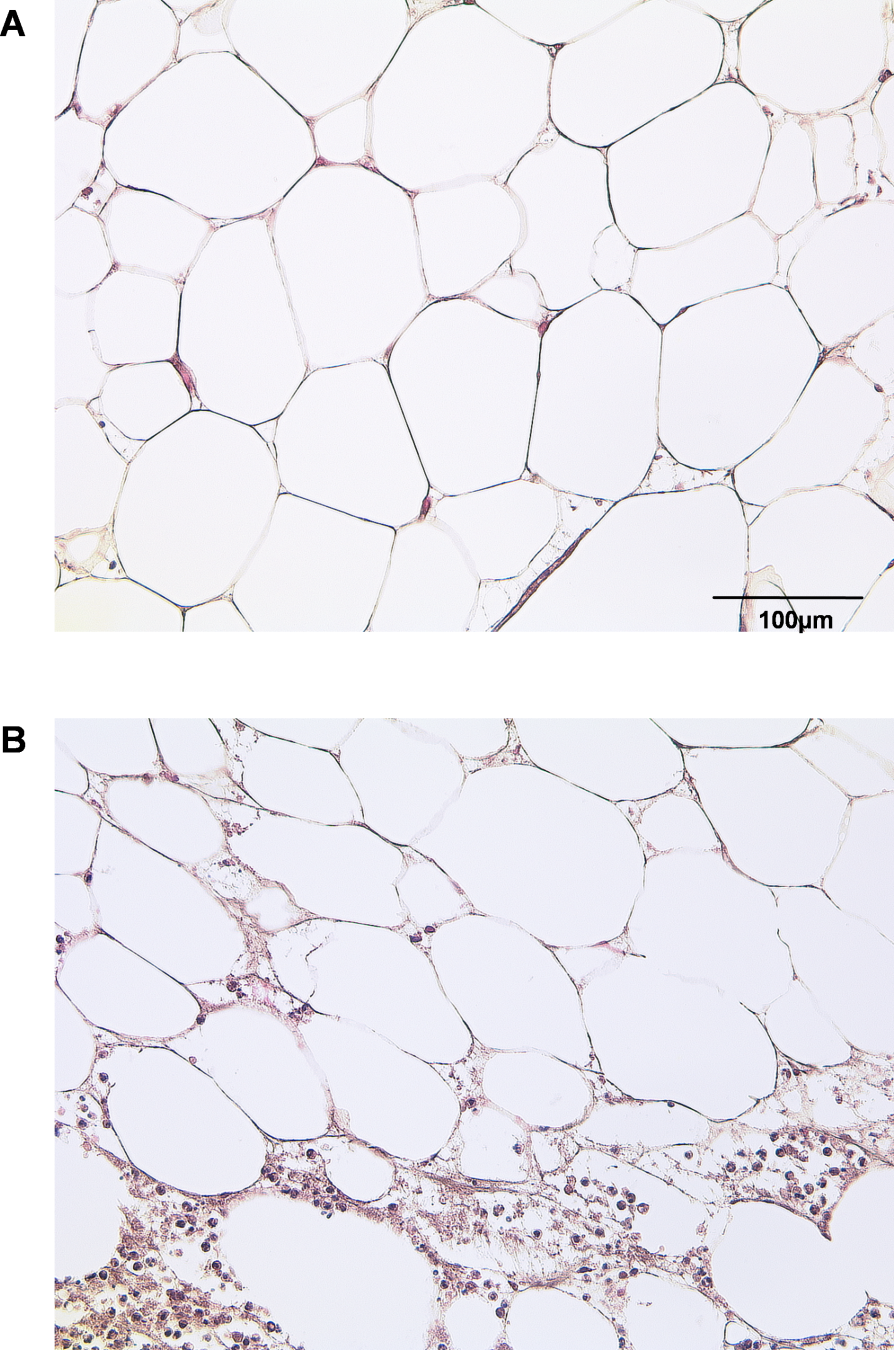
Histological section of HAT and IAT. Histological sections from healthy adipose tissue (HAT) and inflammatory adipose tissue (IAT) samples adjacent to acute wound healing disorders were stained by hematoxylin/eosin (400× maginification). A HE staining of HAT B HE staining of IAT.

By HE staining we sought to illustrate the state of native adipose tissue under normal and inflammatory conditions (Fig. 1). Samples of IAT show a significantly increased infiltration of inflammatory cells when compared to HAT.

### mRNA expression of M1 markers are increased in IAT

**Figure 2:**
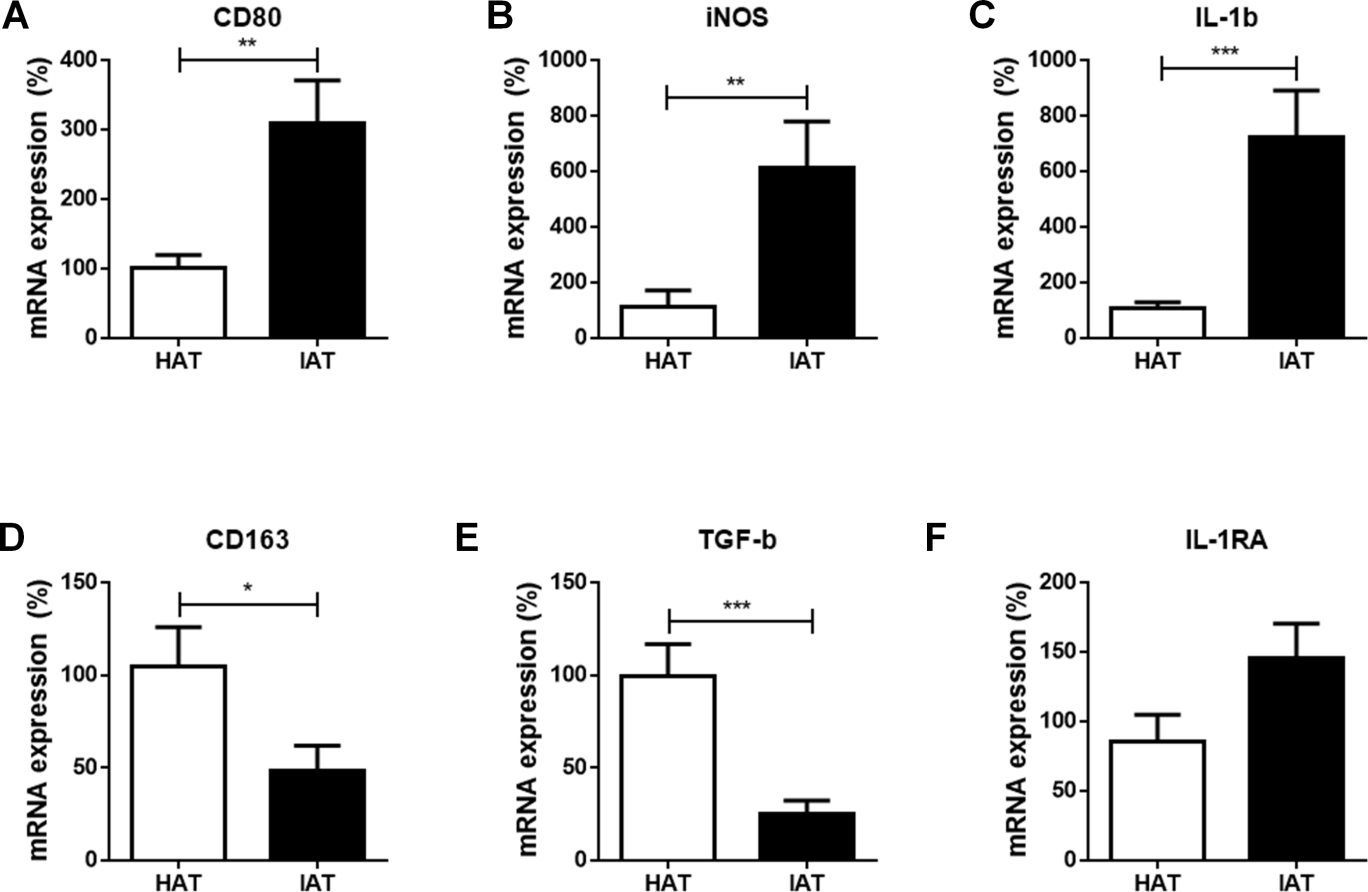
Messenger RNA expression levels of M1- and M2 markers in native HAT and IAT. Messenger RNA was extracted from healthy adipose tissue (HAT, *n* = 20) and inflammatory adipose tissue (IAT, *n* = 20) samples adjacent to acute wound healing disorders . Expression of common pro-inflammatory M1 and anti-inflammatory M2 macrophage markers were measured by qRT-PCR. Relative mRNA expression levels are illustrated with mRNA expression of HAT set as 100%. (A–C) mRNA expression of M1 markers CD80, iNOS, and IL-1*β*; (D–F) mRNA expression of M2 markers CD163, TGF-*β*, and IL-1RA. Data are presented as mean mRNA expression ± SEM. Statistically significant differences are indicated by asterisks (^∗^, *p* < 0.05; ^∗∗^, *p* < 0.01; ^∗∗∗^, *p* < 0.001).

At first, we compared the mRNA expression of the common M1 markers cluster of differentiation 80 (CD80), inducible nitric oxide synthase (iNOS), and interleukin-1*β* (IL-1*β*), between IAT and HAT by qRT-PCR. All three M1-related genes were significantly elevated in IAT compared to HAT (CD80: *p* < 0.01; iNOS: *p* < 0.0; IL-1b: *p* < 0.00; Figs.  2A–2C).

### mRNA expression of M2 markers are decreased in IAT

Next, we evaluated the expression of the common M2 markers CD163, transforming growth factor*β* (TGF-*β*), and interleukin-1 receptor antagonist (IL-1RA) in IAT and HAT. CD163 and TGF-*β* mRNA expression were significantly down-regulated in IAT when compared to HAT (CD163: *p* < 0.0; TGF*β*: *p* < 0.00;  Figs. 2D and 2E. IL-1RA levels, however, showed a slight up-regulation although this effect did not reach statistical significance (Fig. 2F).

### M1 ATM populations are increased while M2 ATM populations are decreased in IAT

**Figure 3:**
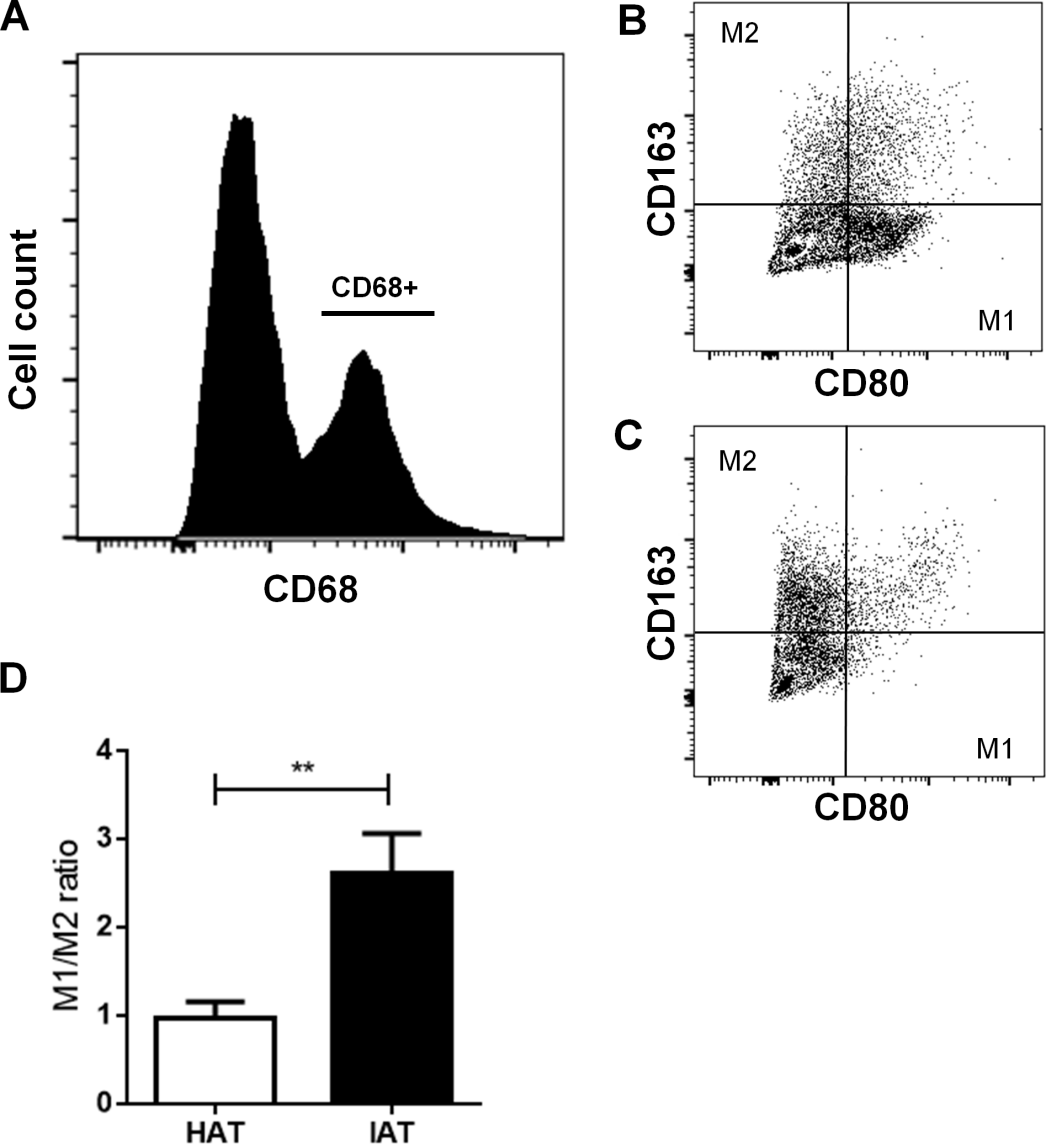
Surface expression of CD80 and CD163 in native HAT and IAT. Stromal vascular fraction (SVF) cells were collected by collagenase digestion of healthy adipose tissue (HAT, *n* = 20) and inflammatory adipose tissue (IAT, *n* = 20) samples and subjected to flow cytometry analysis. (A) ATM were gated by selecting cells which were positive for the pan-macrophage marker CD68. (B) ATMs in IAT show high expression of the M1 macrophage marker CD80 and low expression of the M2 macrophage marker CD163. (C) ATMs in HAT show high expression of CD163 but low expression of CD80. (D) The M1/M2 ratio was calculated as the ratio between CD80+ and CD163+ cells and show an increased M1/M2 ratio in IAT. Data are presented as mean mRNA expression ± SEM. Statistically significant differences are indicated by asterisks (^∗∗^, *p* < 0.01).

After examining the gene expression of M1/M2 markers, we examined more closely M1 and M2 populations by flow cytometry. Freshly isolated SVF cells were stained with the pan macrophage marker CD68, the M1 marker CD80, and the M2 marker CD163. After gating CD68-positive cells (Fig. 3A), the M1/M2 ratio was calculated by dividing the number of CD80 positive cells by CD163 positive cells (Figs. 3B and 3C). IAT showed a significantly increased M1/M2 ratio with a higher number of CD68+/CD80+ M1 ATMs and a lower number of CD68+/CD163+ M2 ATMs than HAT (*p* < 0.01; Fig. 3D).

### Stem cell markers are decreased in IAT

**Figure 4:**
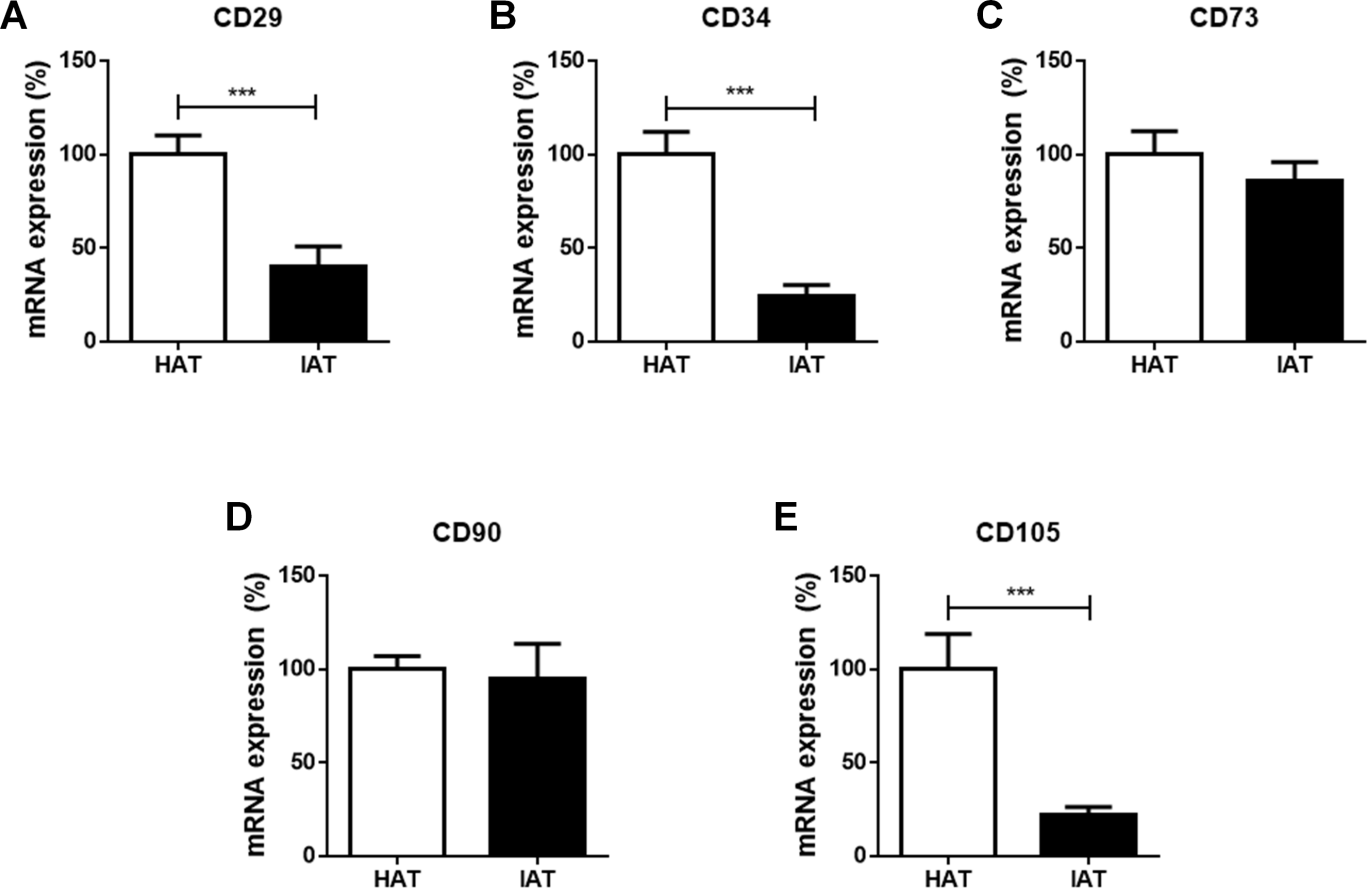
Messenger RNA expression of stem cell markers in native HAT and IAT. Messenger RNA was extracted from healthy adipose tissue (HAT, *n* = 20) and inflammatory adipose tissue (IAT, *n* = 20) samples. Expression of common stem cell markers were measured by qRT-PCR. Relative mRNA expression levels are illustrated with mRNA expression of HAT set as 100%. (A) mRNA expression of CD29 (B) mRNA expression of CD34 (C) mRNA expression of 73 (D) mRNA expression of 90 (E) mRNA expression of CD105. Data are presented in mean mRNA expression ± SEM. Statistically significant differences are indicated by asterisks (^∗∗∗^, *p* < 0.001).

Finally, the mRNA expression of the stem cell markers CD29, CD34, CD73, CD90, and CD105 were investigated in IAT and HAT by qRT-PCR. We observed a significant decrease in the expression of CD29 (*p* < 0.001; Fig. 4A), CD34 (*p* < 0.001; Fig. 4B), and CD105 (*p* < 0.00; Fig. 4E) in IAT when compared to HAT whereas CD90 gene expression (Fig. 4D) was comparable in both groups. CD73 mRNA was slightly reduced in IAT (Fig. 4C) but the difference was statistically not significant.

### Macrophage and stem cell markers are altered independently of the anatomical origin of the samples

To evaluate a possible influence of the anatomical origin of the IAT and HAT samples on our results, we divided samples according to their anatomical harvest side which in general were either the abdomen (Figs. S1 and S2) or the lower extremity (Figs. S3 and S4). As the number of samples that were harvested from anatomical areas other than the abdomen or the lower extremity was too small, respective values were removed from Figs. S1–S4 and not analyzed separately. We found that the harvest side did not markedly influence the outcome of M1/M2 and stem cell marker expression. Matched IAT and HAT samples from the abdomen and the lower extremity both showed an up-regulation of M1 markers in IAT, down-regulation of M2 markers (except for IL-1RA) and stem cell markers CD29, CD34, and CD105 in IAT.

## Discussion

In the present study, we investigated the polarization of ATMs and the expression of ASCs in subcutaneous adipose tissue adjacent to acutely inflamed, non-healing wounds. We found that IAT showed an increase in the mRNA expression of the M1-related genes CD80, iNOS, and IL-*β*, and a down-regulation of the M2-related genes CD163, TGF*β*. The expression of the M2-specific gene IL-1RA was slightly increased in IAT although no statistical significance was reached. The phenotype shift towards the M1 subpopulation was additionally confirmed by flow cytometry, in which a higher M1/M2 ratio was found in IAT. We also observed a marked decrease in the mRNA expression of the stem cell marker CD29, CD34, and CD105 in IAT compared to HAT, while the levels CD73 and CD90 were unaffected. Marker expression was not dependent on the anatomical origin of the samples

As an innate immune response upon tissue damage and pathogens, inflammation orchestrates the clearance of the injurious source and protects the affected tissue (Bashir et al., 2015). Inflammation also is accompanied by the accumulation of inflammatory cells. In our histological sections, that did not discern between cell type or polarization status of macrophages, we could confirm an enhanced infiltration of inflammatory cells into adipose tissue from inflammatory wounds. Monocytes migrate into the wound, differentiate into macrophages, and together with fibroblasts remodel the fibrin matrix into new granulation tissue. Granulation tissue facilitates subsequent wound healing by keratinocytes, and further promotes wound healing by secreting soluble factors that induce cell differentiation (Gurtner et al., 2008). Macrophage depletion from wounds leads to a pathological abruption of wound healing by impaired re-epithelialization, wound granulation, angiogenesis, loss of myofibroblast differentiation, and increased expression of pro-inflammatory cytokines alongside decreased growth factor expression (Jetten et al., 2014). Over the past several years, it has become evident that macrophages possess a phenotypic heterogeneity that allows them to adapt to their environment. The division into pro-inflammatory M1 and anti-inflammatory M2 macrophages with distinct marker profiles, has advanced our understanding of macrophage physiology and is a plausible starting point to interpret tissue responses to inflammatory processes (Sica & Mantovani, 2012). Importantly, macrophage polarization also plays a crucial role in wound healing and it has been reported that M1 and M2 macrophages are found in wounds (Daley et al., 2010). M1 macrophages secrete high amounts of pro-inflammatory cytokines and oxygen and nitrogen radicals that in turn increase the phagocytotic and antimicrobial potency of macrophages (Dale, Boxer & Liles, 2008). M2 macrophages, by contrast, down-regulate pro-inflammatory stimulation by releasing anti-inflammatory cytokines and antagonizing M1 macrophage responses in wounds (Sindrilaru et al., 2011). M2 macrophages are widely considered to be pro-resolving in their actions (Roszer, 2015) and secrete growth factors that activate epithelial cells and facilitate fibroblast differentiation into myofibroblasts (Roberts et al., 1986). M2 macrophages also promote extracellular matrix turnover, clear apoptotic/necrotic cells, debris, and tissue-damaging ECM components, and orchestrate *T*_H_2- and regulatory T cell migration to the wound side (Murray & Wynn, 2011).

To evaluate the macrophage polarization status, we measured the expression of the M1 markers CD80, iNOS, IL-*β*, and the M2 markers CD163, TGF-*β*, and IL-1RA. The M1 and M2 markers used in our study are just a few of a long list of markers that are described in the literature. The surface receptor CD80 delivers an array of different signals that contribute to T-cell activation (Balbo et al., 2001). Wound macrophages were reported to express CD80 that acts as a costimulatory factor for major histocompatibility complex (MHC) I/II molecules in antigen presentation. Together with CD86, CD80 also acts as a prerequisite factor for immune activation in tumors (Olesch et al., 2015). Inducible NOS is an enzyme that catabolizes L-arginine to nitric oxide (NO) which exerts antimicrobial activity (MacMicking, Xie & Nathan, 1997). IL-*β* is a key pro-inflammatory cytokine that is primarily expressed by M1 macrophages. It is up-regulated in activated macrophages and pro-inflammatory macrophages isolated from diabetic wounds (Beuscher, Gunther & Rollinghoff, 1990; Mirza et al., 2013).

CD163 is a receptor primarily expressed by M2 macrophages (Edin et al., 2012). Aside from its homeostatic functions, which include the binding of hemoglobin/haptoglobin complexes, CD163 also has inflammation resolving properties (Fabriek, Dijkstra & Van den Berg, 2005). The cytokine TGF-*β* is characteristically produced by M2 polarized macrophages and has numerous beneficial effects in wound repair (Sporn et al., 1983). It promotes re-epithelialization by mediating the chemotaxis of keratinocytes and endothelial cells, and it supports the translocation of macrophages to the wound (Hameedaldeen et al., 2014). Besides positive effects, TGF-*β* also plays a role in the pathological scarring and fibrosis by regulation of collagen, fibronectin, and proteoglycan deposition (Leask & Abraham, 2004). Hence, the down-regulation of TGF-*β* has to be interpreted with  caution as its exact function in wound repair is multi-faceted and only additional functional studies  may reveal its function in our particular scenario. IL-1RA is an antagonist of the cytokine IL-1 (Dinarello, Simon & van der Meer, 2012) that reduces inflammatory granulation tissue and maintains lamina propria integrity in mice undergoing airway injury (Nicolli et al.,  2015).

M1 and M2 markers as well as stem cell markers were primarily quantified by qPCR with GAPDH serving as a reference gene. However, inconsistent evidence for the reliability of GAPDH as a reference gene is found in the literature with some authors supporting its suitability (Chechi et al., 2012; Gorzelniak et al., 2001; Zhang et al., 2016) whereas others prefer other reference genes (Mehta et al., 2010; Nazari, Parham & Maleki, 2015).

By flow cytometric analysis of the two common M1 and M2 markers CD80 and CD163, we aimed to further characterize ATM subpopulations in HAT and IAT (Badylak et al., 2008). We found that the M1/M2 ratio was significantly increased in IAT when compared to HAT and that few cells expressed both cell surface proteins indicating that the aforementioned markers are appropriate to distinguish M1 and M2 ATMs.

Few studies have investigated the polarization of macrophages in wound healing disorders. Sindrilaru et al. have reported unrestrained M1 macrophage populations in chronic venous leg ulcers as a result of iron overloading that may impede wound healing by perpetuating the chronic inflammatory status (Sindrilaru et al., 2011). In a murine study, Willenborg et al. reported a predominance of M2-genes in the late stage of wound repair whereas M1- and M2-genes were up-regulated in the early stage (Willenborg et al., 2012). Knipper demonstrated that murine wound macrophages showed a prolonged M1 phase and may therefore lead to a chronification of wounds (Knipper, 2013). We also observed an increase of CD80+/CD163- M1 macrophages and an up-regulation of M1-related genes in our study. In contrast to Sindrilaru et al. who collected samples from the wound edges and the skin in chronic ulcers, we focused on ATM harvested from the adjacent subcutaneous tissue in a more acute stage of wound healing that occurs within four weeks after trauma. Our data suggests that not only macrophages in the immediate wound but also ATM from surrounding adipose tissue undergo a phenotype switch towards the M1 to influence wound healing.

The ATM shift towards the M1 phenotype in IAT may be deleterious to wound healing by promoting inflammation. The interpretation, however, that an excess of M1 cells and the lack of anti-inflammatory M2 macrophages is the main source of non-healing wounds is likely too simplistic. Jetten and colleagues showed that the injection of ex-vivo polarized M2 macrophages into cutaneous wounds did not result in improved wound healing in wildtype mice and even delayed wound healing in diabetic mice (Jetten et al., 2014). In our study, the unexpected increase of the M2-marker IL-1RA in IAT suggests that macrophage polarization is not static but a dynamic process that changes during the maturation of wounds (Daley et al., 2010).

ASCs are multipotent mesenchymal stem cells with the ability to self-renew, proliferate, and differentiate into various cell lineages . (Anderson, Gage & Weissman, 2001). ASCs express surface markers that are similar to BMSCs. The minimal criteria of cultured mesenchymal stem cells as defined by the International Society for Cellular Therapy is the expression of CD73, CD90, and CD105 (and absence of CD11b/CD14, CD19/CD79, CD45, HLA-DR) (Dominici et al., 2006). CD29 and CD34 are additional surface markers that are consistently expressed in mesenchymal stem cells (Davies et al., 2015; Suga et al., 2009) IAT showed significant reduction in CD29, CD34, and CD105 compared to HAT. CD29 is a highly preserved surface marker for mesenchymal stem cells and together with CD90 employed to isolate cells with adipogenic differentiation potential (Hayashi et al., 2008; Zavan et al., 2007). Suga et al. reported that CD34+ ASCs show a higher proliferation rate and that CD34 may correlate with the “stemness” of ASCs (Suga et al., 2009). CD105+ ASCs display higher differentiation, proliferation, and colony forming capacity (Lv et al., 2012). Together, the reduction of CD29, CD34, and CD105 expression may be an important contributor to impaired wound healing due to the diminished proliferation and differentiation ability of ASCs.

Many authors have hypothesized that the adipose tissue properties may differ among the anatomical regions of the human body (Aksu et al., 2008; Buschmann et al., 2013; Engels et al., 2013; Fraser et al., 2007; Jurgens et al., 2008; Lacy & Bartness, 2005; Liehn et al., 2013; Padoin et al., 2008; Rohrich, Sorokin & Brown, 2004; Ullmann et al., 2005). However, we could not find any influence of the origin on the change of macrophage or stem cell markers suggesting that the up-regulation of M1 macrophages and down-regulation of stem cell markers is a general change adipose tissue in non-healing wounds undergo.

One way to address the loss of CD29+, CD34+, CD105+ ASCs and counteract the ATM shift towards the pro-inflammatory M1 phenotype in acute wound healing disorders is the transfer of healthy adipose tissue with physiological ASCs and ATMs to the wound site. Fat grafting (the transfer of adipose tissue after processing steps such as centrifugation and decantation) and stem cell therapy (the transfer of SVF cells after collagenase digestion or *in vitro* cultured ASCs) are established and simple methods for the treatment of non-healing wounds such as ulcers, and burn injuries (Brongo et al., 2012; Bruno et al., 2013; Cervelli, Gentile & Grimaldi, 2009; Han, Kim & Kim, 2010; Klinger et al., 2010; Klinger et al., 2008; Zografou et al., 2013). While the differentiation of progenitor cells and delivery of soluble factors were proposed to be key mechanisms by which fat grafts elicit their curative effect, our study indicates that altered populations of ASCs and skewed ATM phenotypes also may contribute to therapeutically beneficial effects.

We have to acknowledge limitations of our study. While our study describes the pro-inflammatory state of macrophages in the adjacent adipose tissue during acute wound inflammation, it does not explain whether local M1 ATMs are a source for the inflammation process or a response to the continuous inflammation. To confirm the underlying mechanisms and consequences of ATM polarization in wounds, additional experimental studies are necessary. Our qRT-PCR and flow cytometry analysis merely examined relative ratios of M1 an M2 markers and stem cell markers. However, it also may be interesting to compare absolute numbers, e.g., number of ATMs, M1 and M2 macrophages, and ASCs per gram adipose tissue, in the future.

## Conclusion

In conclusion, we have shown that adipose tissue that is adjacent to poorly healing wounds exhibits a reduced expression of the stem cell markers CD29, CD34, and CD105 and contains macrophages that are skewed towards the pro-inflammatory M1 phenotype. As our study is of pure descriptive nature and did not include mechanistic aspects, further studies are required to identify possible interactions between ATMs, ASCs, and other cells that are involved in the wound healing process. Futhermore, the use of the often suggested therapeutic approach of autologous fat grafting and ASC therapy for wound repair has to be evaluated in greater detail in the future.

##  Supplemental Information

10.7717/peerj.2824/supp-1 Figure S1 Messenger RNA expression levels of M1- and M2 markers in native HAT and IAT from the abdomen Messenger RNA from IAT and HAT harvested from the abdomen were analyzed. Expression of the M1-specific markers CD80 (A), iNOS (B), and IL-1b (C) and the M2-specific markers CD163 (D), IL-1RA (E), and TGF-*β* (F) were measured by qRT-PCR. Data are presented as mean ± SEM, two-tailed Student’s *t*-test. Statistically significant differences are indicated by asterisks (^∗^*p* < 0.05, ^∗∗^*p* < 0.01, ^∗∗∗^*p* < 0.001).

10.7717/peerj.2824/supp-2 Figure S2 Messenger RNA expression levels of stem cell markers in native HAT and IAT from the abdomen Messenger RNA from IAT and HAT harvested from the abdomen were analyzed. Expression of the mesenchymal stem cell markers CD29 (A), CD34 (B), CD73 (C), CD90 (D), and CD105 (E), were measured by qRT-PCR. Data are presented as mean ± SEM, two-tailed Student’s t-test. Statistically significant differences are indicated by asterisks (^∗∗∗^*p* < 0.001).

10.7717/peerj.2824/supp-3 Figure S3 Messenger RNA expression levels of M1- and M2 markers in native HAT and IAT from the lower extremity Messenger RNA from IAT and HAT harvested from the lower extremity were analyzed. Expression of the M1-specific markers CD80 (A), iNOS (B), and IL-1b (C) and the M2-specific markers CD163 (D), IL-1RA (E), and TGF-*β* (F) were measured by qRT-PCR. Data are presented as mean ± SEM, two-tailed Student’s *t*-test. Statistically significant differences are indicated by asterisks (^∗^*p* < 0.05, ^∗∗^*p* < 0.01, ^∗∗∗^*p* < 0.001).

10.7717/peerj.2824/supp-4 Figure S4 Messenger RNA expression levels of stem cell markers in native HAT and IAT from the lower extremity Messenger RNA from IAT and HAT harvested from the lower extremity were analyzed. Expression of the mesenchymal stem cell markers CD29 (A), CD34 (B), CD73 (C), CD90 (D), and CD105 (E), were measured by qRT-PCR. Data are presented as mean ± SEM, two-tailed Student’s t-test. Statistically significant differences are indicated by asterisks (^∗∗∗^*p* < 0.001).

10.7717/peerj.2824/supp-5 Data S1 Raw data of the qPCR and FACS analysis presented in %

## Additional Information and Declarations

### Data Availability

The following information was supplied regarding data availability: The raw data has been supplied as Supplemental Datasets.
