# Supplementary figures and images for: Characterization of adipose tissue macrophages and adipose-derived stem cells in critical wounds
Source: PeerJ. 2017 Jan 4;5:e2824. doi: 10.7717/peerj.2824 (PMC5217526; doi:10.7717/peerj.2824)

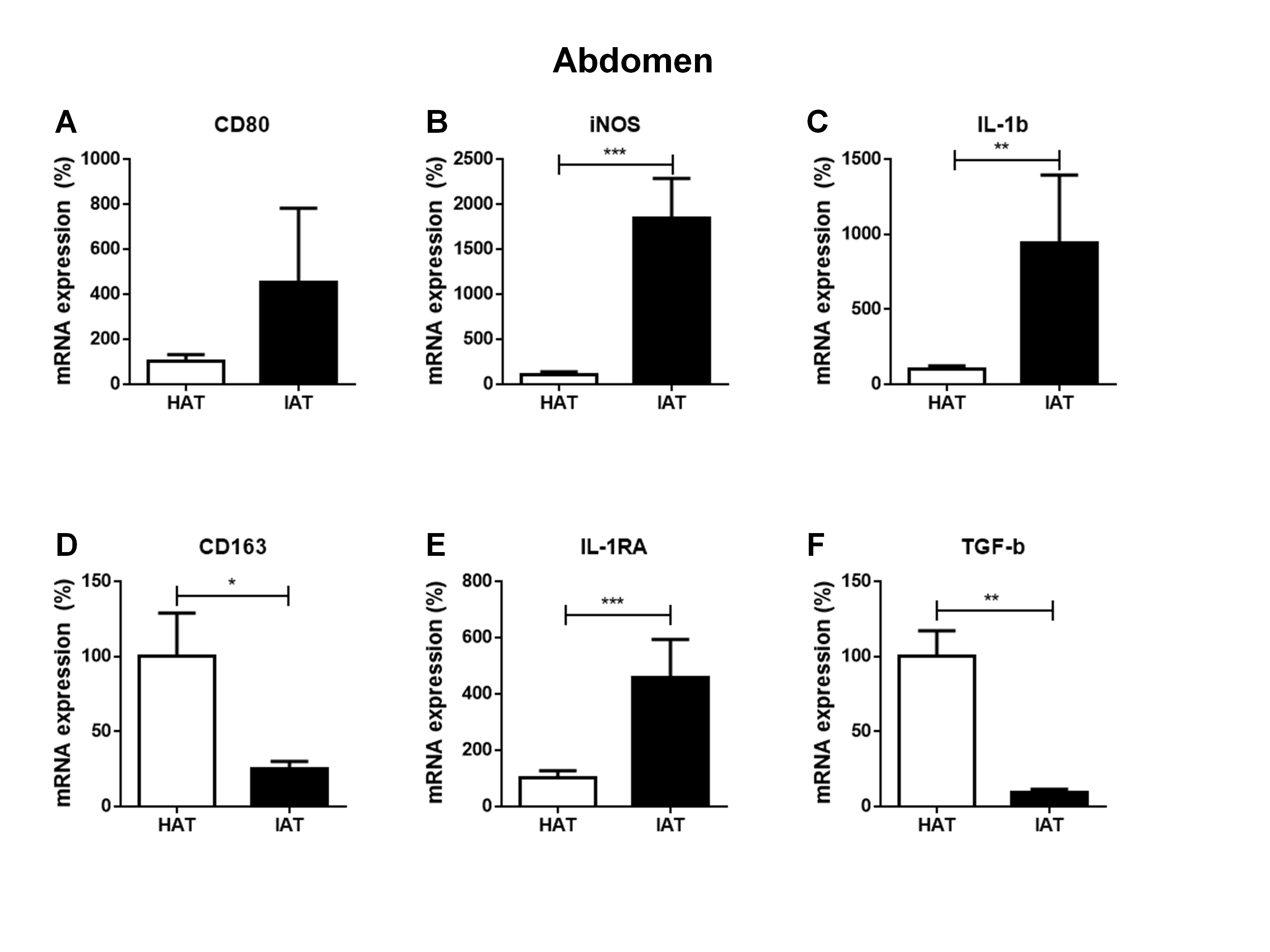

Supplement: Figure S1 — Messenger RNA from IAT and HAT harvested from the abdomen were analyzed. Expression of the M1-specific markers CD80 (A), iNOS (B), and IL-1b (C) and the M2-specific markers CD163 (D), IL-1RA (E), and TGF-β (F) were measured by qRT-PCR. Data are presented as mean ± SEM, two-tailed Student’s t-test. Statistically significant differences are indicated by asterisks (∗p < 0.05, ∗∗p < 0.01, ∗∗∗p < 0.001). [file peerj-05-2824-s001.png]

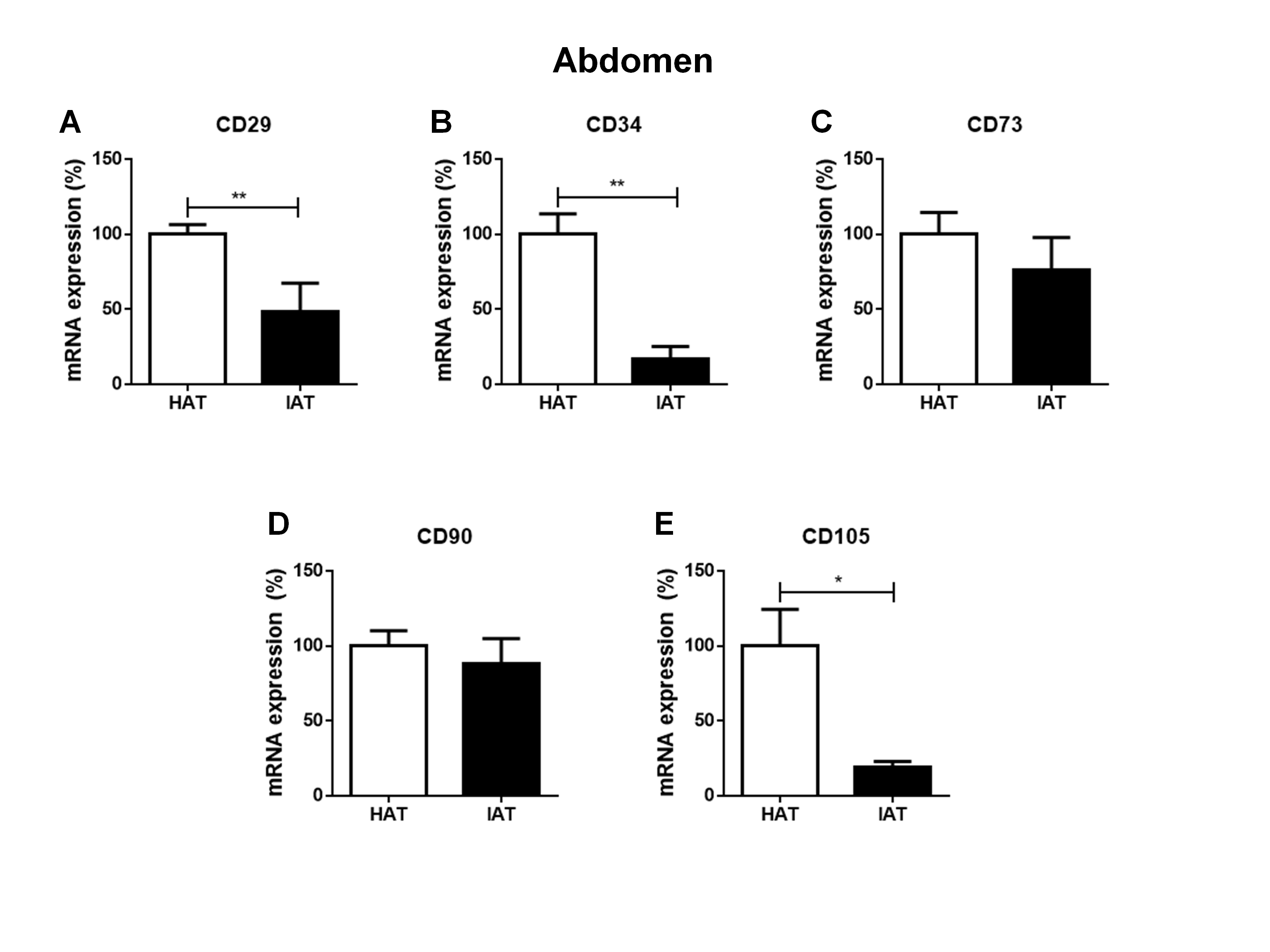

Supplement: Figure S2 — Messenger RNA from IAT and HAT harvested from the abdomen were analyzed. Expression of the mesenchymal stem cell markers CD29 (A), CD34 (B), CD73 (C), CD90 (D), and CD105 (E), were measured by qRT-PCR. Data are presented as mean ± SEM, two-tailed Student’s t-test. Statistically significant differences are indicated by asterisks (∗∗∗p < 0.001). [file peerj-05-2824-s002.png]

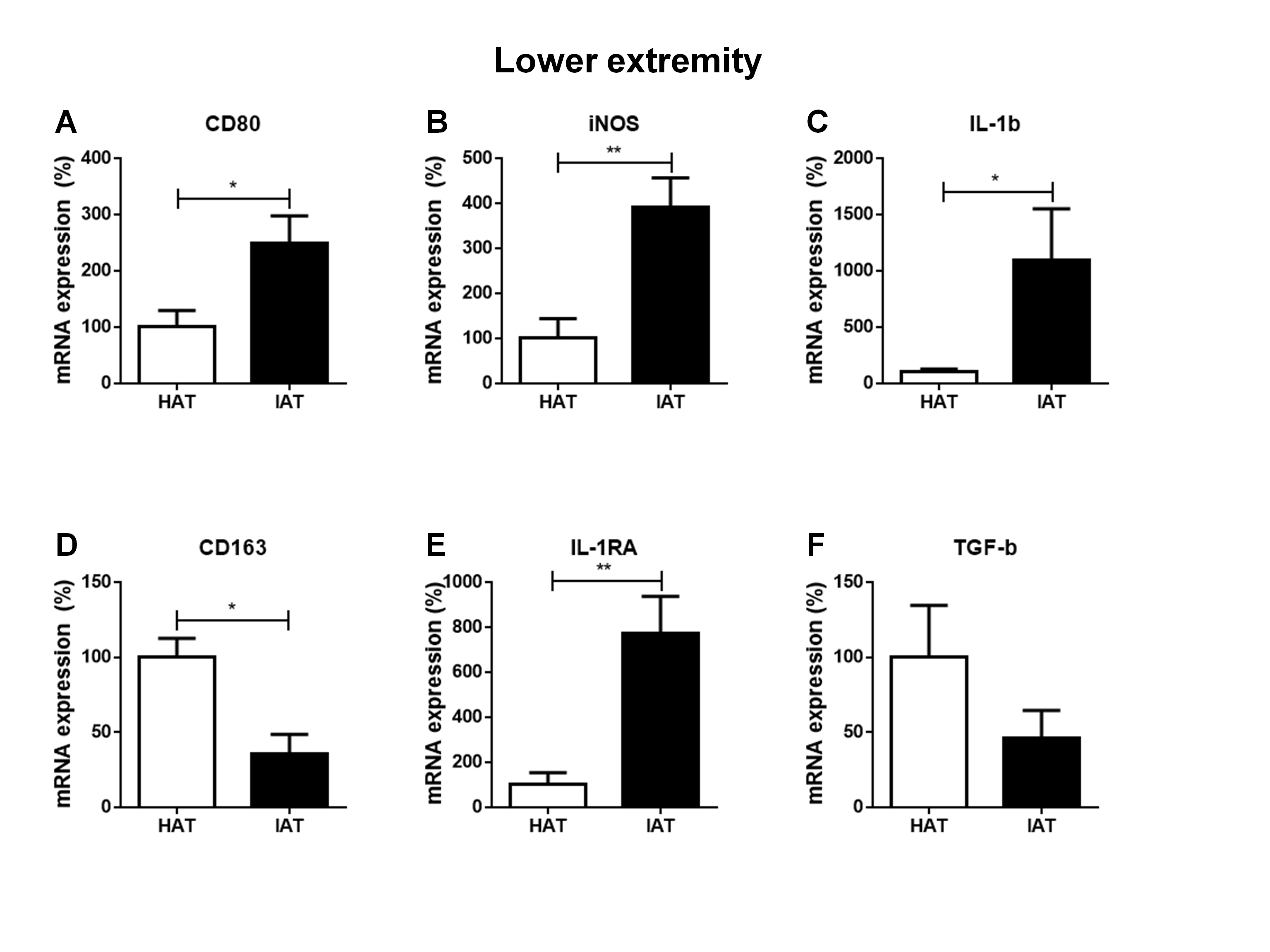

Supplement: Figure S3 — Messenger RNA from IAT and HAT harvested from the lower extremity were analyzed. Expression of the M1-specific markers CD80 (A), iNOS (B), and IL-1b (C) and the M2-specific markers CD163 (D), IL-1RA (E), and TGF-β (F) were measured by qRT-PCR. Data are presented as mean ± SEM, two-tailed Student’s t-test. Statistically significant differences are indicated by asterisks (∗p < 0.05, ∗∗p < 0.01, ∗∗∗p < 0.001). [file peerj-05-2824-s003.png]

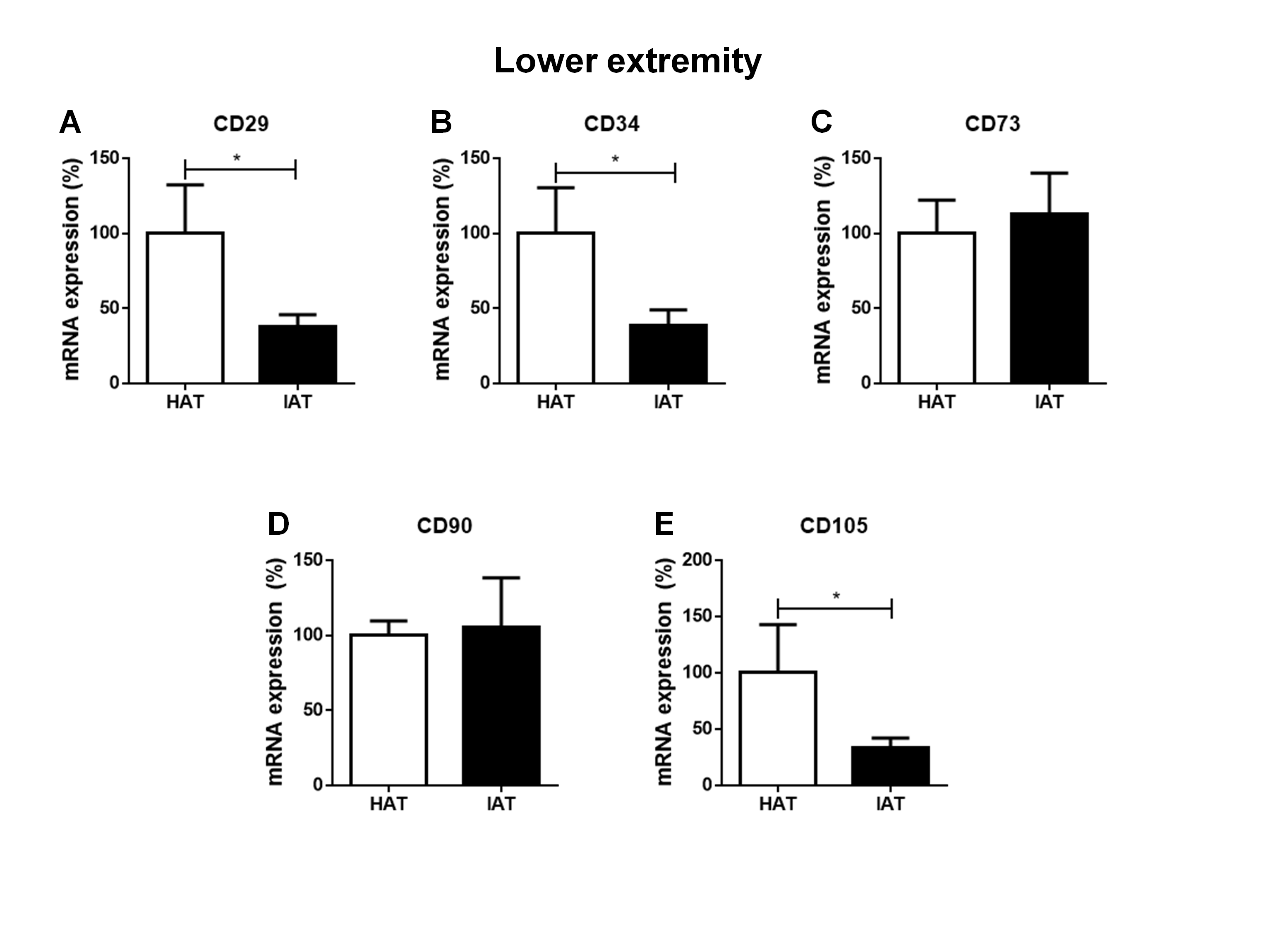

Supplement: Figure S4 — Messenger RNA from IAT and HAT harvested from the lower extremity were analyzed. Expression of the mesenchymal stem cell markers CD29 (A), CD34 (B), CD73 (C), CD90 (D), and CD105 (E), were measured by qRT-PCR. Data are presented as mean ± SEM, two-tailed Student’s t-test. Statistically significant differences are indicated by asterisks (∗∗∗p < 0.001). [file peerj-05-2824-s004.png]
